# Ultrasound-Guided Airway Evaluation: Is It a Game Changer for Predicting Difficult Endotracheal Intubation?

**DOI:** 10.7759/cureus.52557

**Published:** 2024-01-19

**Authors:** Sri Vidhya, Bhanu P Swain, Anbesh Dash, Amlan Swain, Seelora Sahu

**Affiliations:** 1 Anaesthesiology, Tata Main Hospital, Jamshedpur, IND; 2 Anaesthesiology, Manipal Tata Medical College, Manipal Academy of Higher Education, Manipal, IND; 3 Anaesthesiology, Vikash Multi Specialty Hospital, Bargarh, IND

**Keywords:** difficult airway, endotracheal, intubation, laryngoscopy, ultrasound, preoperative assessment, airway

## Abstract

Introduction: Airway ultrasound has recently promised to be a valuable addition to preoperative airway assessment tools. This study was undertaken to determine the efficacy of ultrasound-guided measurement of soft tissue thickness (STT) at various levels of the anterior neck in predicting difficult airways in an eastern Indian population.

Objective: The primary objective was to find out the sensitivity, specificity, positive predictive value (PPV), and negative predictive value (NPV) of anterior neck soft tissue thickness at the level of the hyoid (STT-Hyoid) and vocal cords (STT-VC), distance from the skin to the epiglottis midway (DSEM), and the ratio of the depth of the pre-epiglottic space (Pre-E) to the distance from the epiglottis to the mid-point of the vocal cords (E-VC). The secondary objective was to develop a scoring system using these parameters.

Materials and methods: One hundred eighty-eight patients aged 18-65 years who received general anesthesia and endotracheal intubation for surgery were included in the study. Anterior neck soft tissue thickness measurements were done preoperatively using ultrasound. The actual difficulty of the airway was graded by the anesthesiologist while performing endotracheal intubation using the intubation difficulty scale (IDS).

Results: The incidence of a difficult airway (IDS > 5) was 9.04%. The STT-Hyoid and STT-VC had a moderate correlation with IDS. The DSEM and Pre-E/E-VC ratios had a weak correlation with IDS. For difficult airway prediction, the cutoff points of STT-Hyoid and STT-VC were 7.95 mm and 24.25 mm, respectively. The combined cutoff measurements of STT-Hyoid and STT-VC (29.95 mm) were better predictors of difficult airway.

Conclusion: Preoperative airway ultrasound examination measuring the soft tissue thickness at the hyoid and vocal cord levels is an effective modality in predicting a difficult airway. However, further studies are needed to validate this finding in populations of varied ethnicity and demographic distribution.

## Introduction

Preprocedural airway examination is a routine step before laryngoscopy and endotracheal intubation. It helps identify difficult airways preoperatively and allows the anesthesiologist to plan and be prepared to secure the airway with desired equipment and support. Physical airway assessment methods such as the Mallampati classification, inter-incisor distance, thyromental distance, atlanto-occipital joint extension, and upper lip bite test are commonly used in the preoperative setting. All the above methods have inherently low sensitivities with high variability and hence not always reliable in predicting difficult airways [1]. Unexpected difficult intubations occur in about 1%-8% of cases, despite being predicted as easy airways by these methods [2].

Moreover, physical screening methods are not feasible in unconscious patients. These are often subjective and are prone to interobserver variability. Hence, their diagnostic accuracy remains inadequate, and the search is on for a simple and reliable airway assessment tool.

Ultrasound for airway examination is an attractive alternative to physical assessment tools as it is safe and noninvasive and does not use radiation. The advantage of ultrasound is that it is a point-of-care assessment tool, does not require active patient participation, and can be performed in unconscious patients [3,4]. Ultrasound-guided measurement of soft tissue thickness (STT) at the anterior neck at different levels (hyoid bone, thyrohyoid membrane, and vocal cords) has been suggested to be beneficial in screening difficult airways [5-7]. The rationale behind it is that an increased anterior neck STT could impair the forward movement of the pharyngeal structures, and it will lead to poor exposure of the vocal cords by affecting the upward and forward lift of the laryngoscope blade. Some studies have found a positive correlation between an increased anterior neck STT and airway difficulty, while others failed to find any association [5-9]. Another group of researchers suggested that a high pre-epiglottic space (Pre-E) and a low epiglottis to vocal cord distance (E-VC) are markers of difficult laryngoscopy [10]. However, subsequent studies did not obtain similar results [9]. Ultrasound airway assessment parameters are prone to ethnic and demographic variability [7]. Hence, there is a need to study these parameters in diverse populations to validate it as an effective airway assessment tool.

The current study was undertaken to determine the utility of ultrasound-guided airway assessment in evaluating difficult airways in patients admitted to an eastern Indian hospital. The following parameters were considered: anterior neck soft tissue thickness at the level of the hyoid (STT-Hyoid) and vocal cords (STT-VC), distance from the skin to the epiglottis midway (DSEM), and the ratio of the depth of the pre-epiglottic space (Pre-E) to the distance from the epiglottis to the mid-point of the distance between the vocal cords (E-VC) (Pre-E/E-VC). The primary objective of the study was to find out the sensitivity, specificity, positive predictive value (PPV), and negative predictive value (NPV) of airway ultrasound assessment parameters (STT-Hyoid, STT-VC, DSEM, and Pre-E/E-VC) for predicting a difficult airway quantified by intubation difficulty scale (IDS) [11]. The secondary objective was to develop a scoring system using these parameters.

## Materials and methods

A prospective single-blinded (observer-blinded) study was conducted in a tertiary care hospital in eastern India. The study included 188 patients after obtaining informed consent from all participants. The study received approval from the Institutional Ethics Committee of Tata Main Hospital, Jamshedpur, India. The approval number is TMH/IEC/NOV/26/2018. Patients between the ages of 18 years and 65 years and with American Society of Anesthesiologists (ASA) physical status classification grade I to II undergoing surgery under general anesthesia with airway secured by endotracheal intubation (orotracheal route) were included in the study. Patients requiring rapid sequence intubation and patients with cervical spine pathology, head and neck pathology, and large thyroid swellings were excluded. The study has been registered in the Clinical Trial Registry of India under registration number CTRI/2020/03/024324.

**Figure 1:**
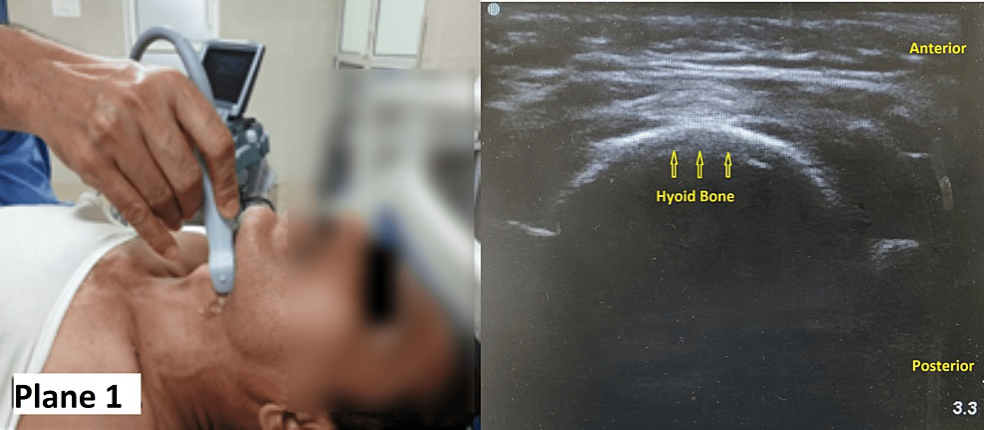
Measurement of the STT-Hyoid Note the position of the ultrasound probe on the neck (Plane 1) and the corresponding image STT-Hyoid: soft tissue thickness at the level of the hyoid

A thorough pre-anesthetic checkup including a detailed systemic examination, relevant investigations, and an airway examination by ultrasound was done in all the patients included in the study. Ultrasound of the airway was performed using a Sonosite M-Turbo Ultrasound System (FUJIFILM Sonosite Inc., Bothell, WA) with an HFL 38/13-6 MHz transducer. All patients were placed in a supine position with a maximal head extension, and the probe was placed at the junction of the neck and floor of the mouth (Plane 1) to measure the soft tissue thickness at the level of the hyoid bone (STT-Hyoid) (Figure 1).

**Figure 2:**
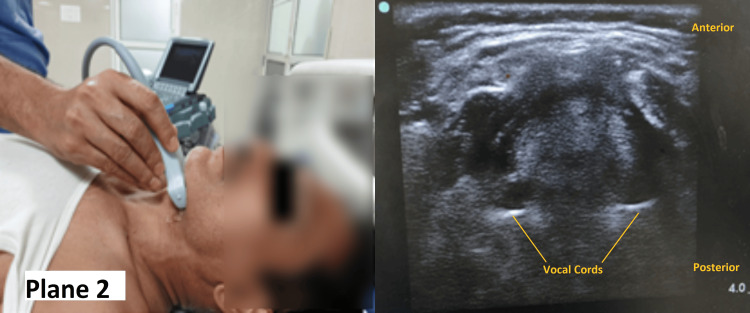
Measurement of the STT-VC Note the position of the ultrasound probe on the neck (Plane 2) and the corresponding image STT-VC: soft tissue thickness at the level of the vocal cord

The probe was then placed at the level of the thyroid cartilage (Plane 2) to measure the soft tissue thickness at the level of vocal cords (STT-VC) (Figure 2). The soft tissue thickness at the level of the epiglottis was measured midway between Plane 1 and Plane 2 (DSEM).

**Figure 3:**
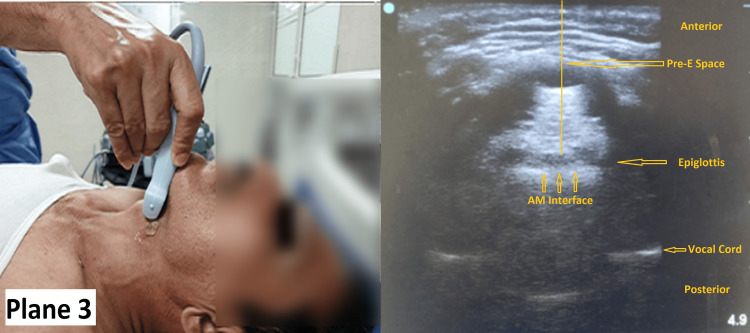
Measurements of Pre-E and E-VC Note the position of the ultrasound probe on the neck (Plane 3) and the corresponding image Pre-E: pre-epiglottic space, E-VC: epiglottis to vocal cord distance, AM: air mucosal

Then, without changing the position of the probe in Plane 1, the ultrasound probe was tilted in the transverse plane from the cephalad to the caudad direction. In this view (Plane 3), the epiglottis was visible as a hypoechoic curvilinear structure. Its anterior border was demarcated by the hyperechoic pre-epiglottic space (PES) and its posterior border by a bright linear air mucosal interface. The posterior-most part of the two vocal folds with arytenoids appeared as hyperechoic lateral V-shaped structures facing away from each other (Figure 3). Patients were instructed to protrude their tongue or perform swallowing to aid in visualizing the epiglottis. Identification of the vocal cords was facilitated by observing their linear movement during quiet breathing or phonation. The depth of the pre-epiglottic space (Pre-E) and the distance from the epiglottis to the mid-point of the distance between the vocal folds (E-VC) was measured. Finally, the ratio of Pre-E and E-VC measurements was calculated and recorded. One of the investigators (Bhanu P. Swain) trained in airway ultrasound conducted the preoperative airway assessment in all patients.

All the study patients received general anesthesia and intermittent positive pressure ventilation after securing the airway with endotracheal intubation by direct laryngoscopy. In the operative room, standard monitors were attached, intravenous access was secured, and after preoxygenation with 100% oxygen for three minutes, anesthesia was induced with intravenous administration of fentanyl 2 µgm/kg and propofol 2.5 mg/kg. After confirming loss of response to verbal command, vecuronium 0.1 mg/kg was given intravenously to facilitate endotracheal intubation. Direct laryngoscopy and endotracheal intubation with an appropriately sized cuffed endotracheal tube was done after the train-of-four (TOF) count became zero in the adductor pollicis muscle. Successful endotracheal intubation was confirmed by capnography and chest auscultation. Anesthesia was maintained with isoflurane in a 50:50 mixture of oxygen and nitrous oxide. All laryngoscopies were performed by another coinvestigator (Sri Vidhya) unaware of the preoperative airway assessment of the patients. The same anesthesiologist noted down the glottis view (Cormack-Lehane (CL) grading) and other parameters of the intubation difficulty scale (IDS). Any patient with failed endotracheal intubation with direct laryngoscopy was excluded from the study.

The following parameters were observed and recorded in all patients: demographic data (age, sex, body mass index (BMI), and ASA grade), ultrasound airway assessment parameters (STT-Hyoid, STT-VC, DSEM, and ratio of Pre-E/E-VC), and intubation difficulty scale (IDS) [11]. An IDS score of less than or equal to 5 is considered easy laryngoscopy and intubation (easy airway) and a score of more than 5 is considered difficult laryngoscopy and intubation (difficult airway) (Table 1).

**Table 1:** Intubation difficulty scale

Score	Description	Points
N1	The number of supplementary attempts (an attempt is defined as one advancement of the tube in the direction of the glottis during direct laryngoscopy)	1 point each
N2	The number of supplementary operators (number of additional persons directly attempting intubation)	1 point each
N3	The number of alternative techniques used	1 point each
N4	Glottic exposure as defined by the Cormack-Lehane grade minus one; grade I (N4 = 0): complete visualization of the vocal cords, grade II (N4 = 1): visualization of the inferior portion of the glottis, grade III (N4 = 2): visualization of only the epiglottis, grade IV (N4 = 3): no visualization of the glottis or epiglottis	0-3 points
N5	The lifting force applied during laryngoscopy; N5 = 0 if little effort was necessary and N5 = 1 if subjectively increased lifting force was necessary	0-1 points
N6	The necessity of applied external laryngeal pressure to optimize the glottis exposure; N6 = 0 if no external pressure was applied and N6 = 1 if external laryngeal pressure was necessary	0-1 points
N7	Position of the vocal cords; N7 = 0 if vocal cords were in abduction and N7 = 1 if vocal cords were in adduction	0-1 points

**Figure 4:**
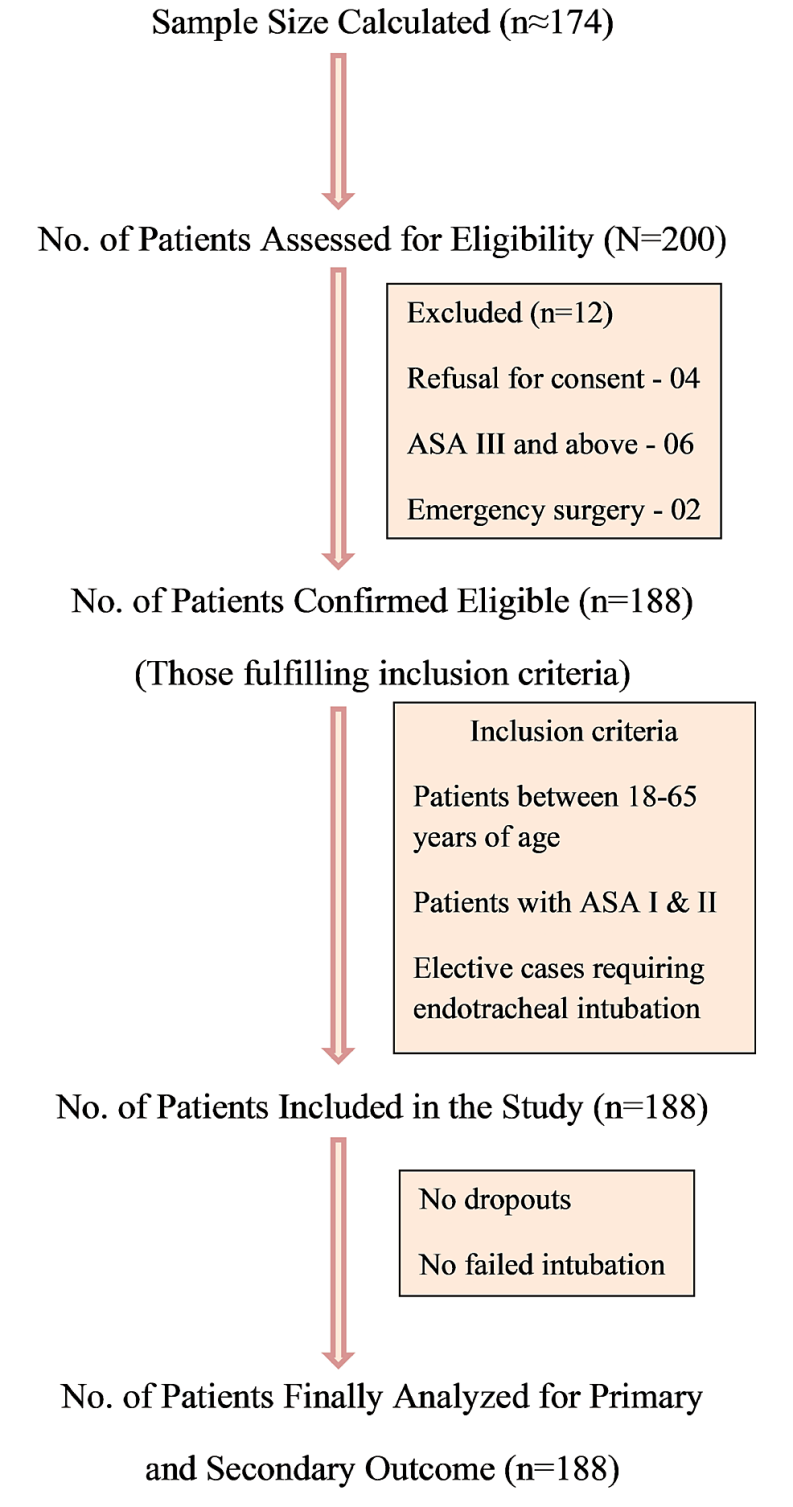
Participant flow diagram ASA: American Society of Anesthesiologists

The sample size estimation was based on the incidence of difficult airways in the Indian population (IDS > 5) found in one of the recent studies [12]. The calculated sample size was 174. To compensate for dropouts (roughly 5%), 188 consecutive patients admitted for elective surgery were included in the study. The participant flow diagram is shown in Figure 4.

The Statistical Package for the Social Sciences (SPSS) version 23.0 was used for statistical analysis. Continuous variables were presented as mean and standard deviation (SD), and categorical variables were presented as absolute numbers and percentages. The comparison of normally distributed continuous variables between the groups was performed using Student’s t-test for two groups and analysis of variance (ANOVA) test for three groups, respectively. Nominal categorical data between the groups were compared using the Chi-squared test or Fisher’s exact test as appropriate. The correlation was checked between IDS and STT-Hyoid, STT-VC, DSEM, and Pre-E/E-VC using Spearman’s rank correlation test. The receiver operating characteristic (ROC) curve was used to summarize the overall accuracy of the sum of STT-Hyoid and STT-VC scores for the identification of intubation difficulty. The ROC plot was obtained by calculating the sensitivity and specificity of every observed cutoff value and plotting sensitivity against specificity. Sensitivity, specificity, positive predictive value (PPV), negative predictive value (NPV), and accuracy were obtained and compared among the cutoff values of the sum of STT-Hyoid and STT-VC.

## Results

The incidence of a difficult airway (IDS > 5) was 9.04%. The demographic parameters are shown in Table 2. There was no significant difference in the age and sex of patients between the easy airway (IDS ≤ 5) and difficult airway (IDS > 5) groups. The mean body mass index (BMI) was statistically higher in the difficult airway group.

**Table 2:** Demographic parameters IDS: intubation difficulty scale, M/F: male/female, BMI: body mass index, SD: standard deviation

Parameters	Easy airway (IDS ≤ 5) (n = 171)	Difficult airway (IDS > 5) (n = 17)	p-value
Gender (M/F) (number (%))	83 (48.5%)/88 (51.5%)	11 (64.7%)/6 (35.3%)	0.2
Age (years) (mean ± SD)	45.01 ± 12.7	49.41 ± 11.38	0.17
BMI (kg/m^2^) (mean ± SD)	26.45 ± 3.03	26.42 ± 4.87	0.001

The values of ultrasound airway assessment parameters (STT-Hyoid, STT-VC, DSEM, and ratio of Pre-E/E-VC) in easy and difficult airway groups are shown in Table 3. The STT-Hyoid and STT-VC measurements were significantly higher in the difficult airway group than in the easy airway group. The DSEM and Pre-E/E-VC ratio measurements in the two groups were not statistically significant, although higher values were recorded in the difficult airway group.

**Table 3:** Ultrasound airway assessment parameters IDS: intubation difficulty scale, STT-Hyoid: soft tissue thickness at the level of the hyoid, STT-VC: soft tissue thickness at the level of the vocal cord, DSEM: distance from the skin to the epiglottis midway, Pre-E/E-VC: ratio of the depth of the pre-epiglottic space (Pre-E) to the distance from the epiglottis to the mid-point of the distance between the vocal cords (E-VC), SD: standard deviation

Parameters	Easy airway (IDS ≤ 5) (n = 171)	Difficult airway (IDS > 5) (n = 17)	p-value
STT-Hyoid (mean ± SD)	7.66 ± 1.37	9.35 ± 2.32	0.00001
STT-VC (mean ± SD)	21.68 ± 4.99	26.29 ± 3.20	0.0002
DSEM (mean ± SD)	14.66 ± 2.15	16.08 ± 2.52	0.1
Pre-E/E-VC (mean ± SD)	1.44 ± 0.30	1.57 ± 0.39	0.8

Table 4 shows the correlation between the ultrasound airway parameters and IDS. There was a significant correlation between STT-Hyoid, STT-VC, and IDS.

**Table 4:** Correlation between ultrasound airway assessment parameters and IDS *Correlation is significant at the 0.01 level (two-tailed). IDS: intubation difficulty scale, STT-Hyoid: soft tissue thickness at the level of the hyoid, STT-VC: soft tissue thickness at the level of the vocal cord, DSEM: distance from the skin to the epiglottis midway, Pre-E/E-VC: ratio of the depth of the pre-epiglottic space (Pre-E) to the distance from the epiglottis to the mid-point of the distance between the vocal cords (E-VC), Sig.: significance level

Ultrasonographic parameters		STT-Hyoid	STT-VC	DSEM	Pre-E/E-VC	IDS
STT-VC	Correlation coefficient	0.513^*^	1.000			
	Sig. (two-tailed)	0.000				
DSEM	Correlation coefficient	0.444^*^	0.292^*^	1.000		
	Sig. (two-tailed)	0.000	0.000			
Pre-E/E-VC	Correlation coefficient	0.432^*^	0.142	0.654^*^	1.000	
	Sig. (two-tailed)	0.000	0.052	0.000		
IDS	Correlation coefficient	0.461^*^	0.514^*^	0.116	0.268^*^	1.000
	Sig. (two-tailed)	0.000	0.000	0.112	0.000	

The cutoff points for the prediction of a difficult airway were calculated for STT-Hyoid, STT-VC, and combinations of both (Table 5). The optimal cutoff points for STT-Hyoid, STT-VC, and the sum of both were 7.95 mm, 24.25 mm, and 29.95 mm, respectively.

**Table 5:** Optimal cutoff points for STT-Hyoid, STT-VC, and the sum of STT (Hyoid + VC) STT-Hyoid: soft tissue thickness at the level of the hyoid, STT-VC: soft tissue thickness at the level of the vocal cord, DSEM: distance from the skin to the epiglottis midway, Pre-E/E-VC: ratio of the depth of the pre-epiglottic space (Pre-E) to the distance from the epiglottis to the mid-point of the distance between the vocal cords (E-VC), PPV: positive predictive value, NPV: negative predictive value

Variables	Cutoff point (mm)	Sensitivity (%)	Specificity (%)	PPV (%)	NPV (%)	Accuracy (%)
STT-Hyoid	7.95	76.50	66.10	18.31	96.58	67.02
STT-VC	24.25	70.60	72.50	20.34	96.12	72.34
STT (Hyoid + VC)	29.95	94.10	48.50	15.38	98.80	52.65

**Figure 5:**
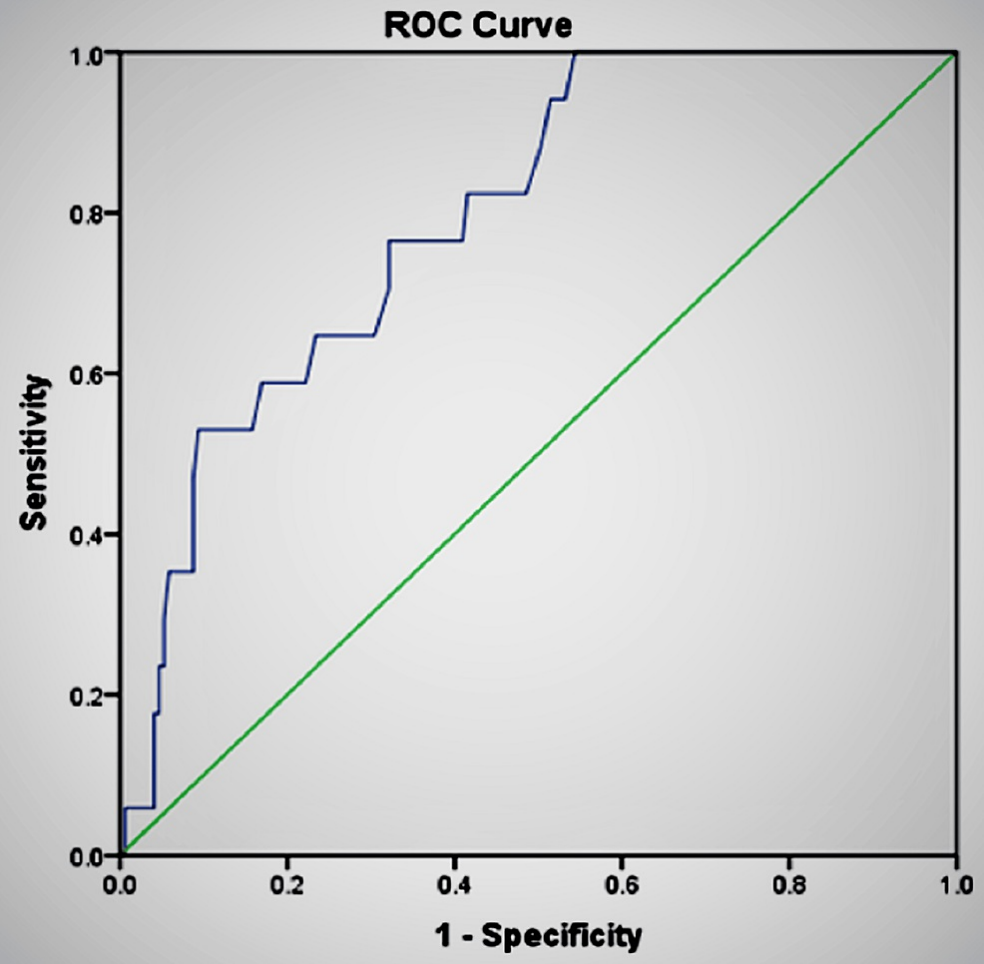
Graphical representation of the correlation of the combined score of STT-Hyoid and STT-VC with IDS using ROC curve ROC: receiver operating characteristic, IDS: intubation difficulty scale, STT-Hyoid: soft tissue thickness at the level of the hyoid, STT-VC: soft tissue thickness at the level of the vocal cord

The receiver operating characteristic curve was used to predict the diagnostic accuracy of the sum score of STT-Hyoid and STT-VC (29.95 mm) with IDS (Figure 5). The area under the curve (AUC) was 0.795 (95% confidence interval (CI): 0.70-0.89).

## Discussion

The current study was conducted to evaluate the efficacy of ultrasound airway assessment parameters in predicting difficult airways. We studied the following parameters: the soft tissue thickness at the level of the hyoid (STT-Hyoid) and vocal cords (STT-VC), thyrohyoid membrane (DSEM), and the ratio of the depth of the pre-epiglottic space to the distance from the epiglottis to the mid-point of vocal cords (Pre-E/ E-VC). The intubation difficulty scale (IDS) was used to assess the difficulty of laryngoscopy and intubation. The study included 174 patients, and they were classified into two groups: easy airway group (IDS ≤ 5) and difficult airway group (IDS > 5) based on the IDS score recorded. The ultrasound airway assessment parameters observed preoperatively were compared between the two groups. Difficult laryngoscopy and intubation were encountered (IDS > 5) in 17 (9.04%) patients. Our study revealed that higher soft tissue thickness at the hyoid bone (SST-Hyoid) and vocal cord (STT-VC) were associated with a difficult airway. The other two parameters (DSEM and Pre-E/ E-VC) did not show any significant correlation with difficult airways.

There were multiple studies done on different populations addressing ultrasound airway assessments and their ability to predict difficult airways. One of the earlier studies by Ezri et al. [5] revealed that pre-tracheal soft tissue at the level of vocal cords (STT-VC) was a good predictor of difficult laryngoscopy in obese patients. Later, Wu et al. [7], Reddy et al. [13], and Jain et al. [14] reported similar findings in their studies. However, a similar study by Komatsu et al. [8] did not show any clinically significant difference in soft tissue thickness (STT) at the vocal cord level between difficult and easy laryngoscopy cases. In our study, 24.25 mm of SST-VC was calculated as the optimal cutoff point for the prediction of the difficult airway with a sensitivity of 70.6% and a specificity of 72.5%. Ezri et al. [5] (range of 24 mm to 32 mm) and Jain et al. [14] (range of 24.03 mm to 27.37mm) reported similar values of STT at the vocal cord in difficult airway patients, whereas Wu et al. [7] (11 mm) and Reddy et al. [13] (2.3 mm) described much lower values in their studies. The difference in the measurements could be attributed to the predominantly obese population in the former two studies (Ezri et al. [5] and Jain et al. [14]). Ezri et al. [5] mentioned that the amount of pre-tracheal soft tissue was the only measure of difficult laryngoscopy in obese individuals. The mean BMI (26.57 ± 03 mm) in our study was in the overweight range, which probably justifies the similarity of our data with Ezri et al. [5] and Jain et al. [14]. However, we did not correlate BMI with STT at the anterior neck and difficult airway as it was beyond the scope of the study.

In our study, STT at hyoid (STT-Hyoid) had a significant correlation with IDS score. We calculated a cutoff value of 7.95 mm (sensitivity: 76.5%, specificity: 66.1%) for difficult airways. Adhikari et al. [6], Wu et al. [7], and Yadav et al. [15] also found a correlation of STT at the hyoid level with Cormack-Lehane grade. Yadav et al. [15] examined STT at the hyoid level both in neutral and sniffing positions and reported a mean value of 7.4 mm (in neutral) and 7.3 mm (in sniffing) in difficult laryngoscopy cases. Adhikari et al. [6] in their pilot study reported a mean value of 16.9 mm (95% CI: 1.19-2.19) (STT-Hyoid), and Wu et al. [7] had a cutoff value of 12.8 mm (sensitivity: 85.7%, specificity: 85.1%) in difficult laryngoscopy cases. The difference in the values of STT-Hyoid predicting difficult airway may be because of the different ethnic origins of the study population.

The soft tissue thickness at the epiglottis level (DSEM) and the Pre-E/ E-VC ratio had a weak correlation with IDS in our study. Similarly, Mohammadi et al. [9] and Reddy et al. [13] found that the Pre-E/E-VC ratio has poor predictive quality of difficult airways. In contrast, studies by Gupta et al. [10] and Rana et al. [16] found a strong positive correlation between the Pre-E/E-VC ratio and CL grade. We observed that obtaining the sonographic view for measuring the Pre-E/ E-VC ratio was time-consuming and technically challenging due to difficulty in locating the air mucosal interface, especially in obese patients. Unlike our study, most studies reported higher STT at the thyrohyoid membrane or skin-to-epiglottis distance (DSEM) associated with poor laryngoscopic view [6,7,15,17,18]. We observed a higher mean value of DSEM in difficult airways than in easy airways, although it was not statistically significant.

In predicting a difficult airway, a parameter with higher sensitivity and moderate specificity is ideal, as it minimizes the false-negative results and reduces the chance of an unanticipated difficult airway. In our study, the cutoff values of SST-Hyoid and STT-VC had only moderate sensitivity of 76.5% and 70.6%, respectively. Hence, to improve the sensitivity of the ultrasound measurement of neck soft tissue thickness in predicting difficult airways, we proposed to combine the cutoff values of STT-Hyoid and SST-VC. It was observed that the combined cutoff value of 29.95 mm increased the sensitivity to 94.1% with a specificity of 48.5%. Moreover, the negative predictive values (NPVs) of SST-Hyoid and STT-VC individually were close to 96%, but with the combined value, NPV increased to 98.8%. This implies that if the combined value of SST-Hyoid and SST-VC is less than 29.95 mm, it effectively rules out the possibility of a difficult airway in 98.8% of cases. The positive predictive value (PPV) of the STT-Hyoid, STT-VC, and the sum of STT-Hyoid and STT-VC were low in our study, which was probably due to the low prevalence of difficult airways in the study population. The receiver operating characteristics (ROC) curve was plotted, and the area under the curve (AUC) was calculated for the combined cutoff value. The AUC was 0.795, which implies that there is a 79.5% chance that the combination will be able to differentiate between an easy and difficult airway.

In the current study, the intubation difficulty scale (IDS) was used to assess the difficulty in endotracheal intubation, which was correlated with preoperative ultrasound-guided airway measurements. All the previous studies have used the laryngoscopic view, measured using the Cormack-Lehane grade for the same [6,7,13-18]. The drawback of the CL grade is that it can only grade the visualization of the glottic opening, and it fails to quantify the ease of tracheal intubation. Conversely, the intubation difficulty scale (IDS) has the advantage of taking into consideration both the laryngoscopic view (Cormack-Lehane grade) and the intubating conditions [19].

At the beginning of the current study, it was hypothesized that the selected four ultrasound airway assessment parameters (STT-Hyoid, STT-VC, DSEM, and Pre-E/ E-VC ratio) would be able to differentiate between an easy and difficult airway. The result showed that only the first two measurements (STT-Hyoid and STT-VC) were sensitive enough to predict difficult laryngoscopy and intubation. The secondary outcome of the study was to formulate an ultrasonographic airway scoring system. We failed to develop a scoring system as only two parameters correlated with a difficult airway. However, our study proved that the combined cutoff values of STT-Hyoid and STT-VC (cutoff value > 29.5 mm) can be a sensitive indicator in differentiating easy from difficult airways.

There are a few limitations in our study. The individual ultrasound airway assessment parameters did not have high sensitivity or specificity to be used as a standalone test to predict difficult airways. By combining the parameters, we achieved high sensitivity, but specificity remained low. The cutoff values of all parameters are derived from the population of eastern India and hence may not be generalizable to other populations. This study has a lower prevalence of difficult airways, which may alter the outcome. The IDS scoring system used in this study has few subjective parameters, which can affect interpretation, especially in the borderline scores (IDS 5 and 6). Operator bias is also possible in preoperative sonographic airway examination, especially in cases of already predicted difficult airway (by physical airway assessment tools). The ultrasound airway examination was done by a single investigator in all cases. The data might be different if more than one person had done the airway assessment due to interobserver variability. Lastly, while doing the soft tissue thickness measurement at the anterior neck by ultrasound, pressure is applied by the ultrasound probe. The outcome may vary if too much pressure is applied. Hence, the ultrasound probe must be placed gently over the skin to obtain accurate measurements.

## Conclusions

Ultrasound-guided airway examination has proven to be a valuable adjunct to the existing clinical preoperative airway screening tools. It promises to revolutionize the way anesthesiologists predict difficult endotracheal intubation preoperatively. Among all the ultrasound airway assessment parameters, an increased soft tissue thickness at the hyoid and the vocal cord level significantly correlates with a difficult airway. Moreover, the combined values of the soft tissue thickness at the hyoid and vocal cord have a better predictive value. Skin-to-epiglottis distance and the ratio of pre-epiglottic space to the distance between the epiglottis and vocal cords do not correlate with difficult airways in our study population. Further studies are warranted to verify our study findings in a diverse population. We recommend the following points for future research: the sonographic airway measurements can be correlated with the clinical airway examinations based on the results of our study as well as findings from other studies and the Pre-E/E-VC ratio can be excluded from the sonographic preoperative airway assessment as it is not only less sensitive to predict difficult airway but also cumbersome to measure.
